# Phenotypic and transcriptional analysis of the osmotic regulator OmpR in *Yersinia pestis*

**DOI:** 10.1186/1471-2180-11-39

**Published:** 2011-02-23

**Authors:** He Gao, Yiquan Zhang, Yanping Han, Lin Yang, Xia Liu, Zhaobiao Guo, Yafang Tan, Xinxiang Huang, Dongsheng Zhou, Ruifu Yang

**Affiliations:** 1 State Key Laboratory of Pathogen and Biosecurity, Beijing Institute of Microbiology and Epidemiology, Beijing 100071, PR China; 2 Department of Biochemistry and Molecular Biology, Jiangsu University School of Medical Technology, Zhenjiang, Jiangsu 212013, PR China

## Abstract

**Background:**

The osmotic regulator OmpR in *Escherichia coli *regulates differentially the expression of major porin proteins OmpF and OmpC. In *Yersinia enterocolitica *and *Y. pseudotuberculosis*, OmpR is required for both virulence and survival within macrophages. However, the phenotypic and regulatory roles of OmpR in *Y. pestis *are not yet fully understood.

**Results:**

*Y. pestis *OmpR is involved in building resistance against phagocytosis and controls the adaptation to various stressful conditions met in macrophages. The *ompR *mutation likely did not affect the virulence of *Y. pestis *strain 201 that was a human-avirulent enzootic strain. The microarray-based comparative transcriptome analysis disclosed a set of 224 genes whose expressions were affected by the *ompR *mutation, indicating the global regulatory role of OmpR in *Y. pestis*. Real-time RT-PCR or *lacZ *fusion reporter assay further validated 16 OmpR-dependent genes, for which OmpR consensus-like sequences were found within their upstream DNA regions. *ompC*, *F*, *X*, and *R *were up-regulated dramatically with the increase of medium osmolarity, which was mediated by OmpR occupying the target promoter regions in a tandem manner.

**Conclusion:**

OmpR contributes to the resistance against phagocytosis or survival within macrophages, which is conserved in the pathogenic yersiniae. *Y. pestis *OmpR regulates *ompC*, *F*, *X*, and *R *directly through OmpR-promoter DNA association. There is an inducible expressions of the pore-forming proteins OmpF, C, and × at high osmolarity in *Y. pestis*, in contrast to the reciprocal regulation of them in *E. coli*. The main difference is that *ompF *expression is not repressed at high osmolarity in *Y. pestis*, which is likely due to the absence of a promoter-distal OmpR-binding site for *ompF*.

## Background

The *ompB *operon consists of the *ompR *and *envZ *genes, whose coding regions overlap by several base pairs; this genetic structure is highly conserved in *Enterobacteriaceae *[1,2]. The inner membrane EnvZ, a histidine kinase, acts as a sensor responding to the elevation of medium osmolarity and undergoes trans-autophosphorylation. The high energy of phosphoryl group is subsequently transferred to the cytoplasmic protein OmpR. The phosphorylated OmpR (OmpR-P) acts as a DNA-binding transcription factor to regulate its target genes. EnvZ also possesses the phosphatase activity to dephosphorylate itself.

Osmotic signals regulate the ratio of kinase/phosphatase activity of EnvZ to modulate the cellular OmpR-P level [1,2]. At low medium osmolarity, OmpR-P levels are also low due to the decreased kinase/phosphatase ratio of EnvZ; on the other hand, at high osmolarity, an elevated OmpR-P level results from the ratio increase. The *ompR *transcription is induced directly by its own gene product in *Salmonella enterica *[3]. OmpR consensus-like sequences are found in the upstream region of *ompR *in *Escherichia coli*, although there are still no reported experimental data for its autoregulation in this bacterium. Upon the elevation of medium osmolarity, cellular OmpR-P levels are likely enhanced by two distinct mechanisms, namely, post-translational phosphorylation/dephosphorylation by EnvZ and transcriptional auto-stimulation.

*Enterobacteriaceae *express at least two major outer membrane (OM) porins, namely, OmpF and OmpC, both of which form transmembrane pore structures and function as ion channel [4-6]. OmpF and OmpC in the cell of *E. coli *form water-filled pores that are poorly selective to cations (so called non-specific porins), thereby allowing the diffusion of low-molecular-weight polar compounds (not over 600 daltons) into the cell to maintain cell permeability. They exist as homotrimers in the OM. The basic structural element of the porin monomer is an ellipsoid in the section cylinder consisting of 16 transmembrane β-strands (so-called β-barrel) connected by short periplasmic and longer 'external' loops [7].

*E. coli *OmpX contains 8-stranded β-barrel, with polar residues on the inside and hydrophobic residues on the outside facing the membrane environment [8]. *Enterobacter aerogenes *OmpX is the smallest known channel protein with a markedly cationic selectivity [6,9,10]. Although several experiments have demonstrated that OmpX plays roles that are similar to those of porin [6,9-12], it is not yet clear whether or not OmpX forms porins on the cell membrane. *E. aerogenes *OmpX forms channels in the lipid bilayer [6]; however, the NMR and crystal structures of OmpX do not show pores [8,13]. The *ompX *expression in *E. coli *[12]*or E. aerogenes *[6] is enhanced during early exposure to environmental perturbations, such as high osmolarity, antibiotics and toxic compounds, that are accompanied by the repressed expression of non-specific porins (OmpF and/or OmpC). Over-expression of OmpX, with a channel structure that is much smaller than that of OmpF and OmpC [6], may stabilize cell OM and balance the decreased expression of the two non-specific porins for the exclusion of small harmful molecules. It is interesting to further investigate the roles of OmpX in modulating OM permeability and adaptability. OmpR consensus-like sequences have been found within the *ompX *upstream region in *E. coli *and *E. aerogenes *[6]; however, the regulation of *ompX *by OmpR has not yet been established experimentally in any bacterium.

As shown in *E. coli *as a model, OmpF and OmpC are reciprocally regulated by medium osmolarity. OmpC is predominant at high osmolarity, while the OmpF expression is repressed; in contrast, the reverse effect is observed at low osmolarity [14]. The reciprocal regulations of Omp36 and Omp35 (OmpF and OmpC-like, respectively) have been established in *E. aerogenes *as well [15]. Tight regulation of porin expression is crucial for bacterial adaptation to environments, which is mediated by a two-component system EnvZ/OmpR [2,16,17]. Likewise, four (tandem F1-F2-F3, and F4) and three (tandem C1-C2-C3) OmpR consensus-like sequences have been determined in the DNA regions upstream of *ompF *and *ompC *in *E. coli*, respectively. At low osmolarity, OmpR-P binds cooperatively to F1-F2 or F1-F2-F3 in order to activate the transcription of *ompF*; meanwhile, it only occupies C1, which is not sufficient to activate the transcription of *ompC*. At high osmolarity, C2-C3 becomes occupied by OmpR-P with the elevated cellular OmpR-P levels, resulting in the *ompC *expression. Moreover, OmpR-P also binds to F4, which is a weak OmpR-P-binding site located 260 bp upstream of F1-F2-F3 to form a loop. In turn, this interferes with the binding of OmpR-P to F1-F2-F3, so as to block the *ompF *transcription.

As a member of the *Enterobacteriaceae *family, the genus *Yersinia *includes three human-pathogenic species, namely, *Y. pestis, Y. pseudotuberculosis*, and *Y. enterocolitica. Y. pestis *causes the deadly plague, while the latter two only cause non-fatal gastroenteric diseases [18]. *Y. pestis *has evolved recently (from the evolutionary point of view) from *Y. pseudotuberculosis *by a process combining gene acquisition, loss and inactivation, while *Y. enterocolitica *represents a far distinct evolutionary lineage [18]. *Yersinia ompF, C*, and *X *contains conservative amino acid residues or domains typical among porins [7,19-21]. However, regulation of porins in *Y. pestis *is not yet fully understood.

Data presented here disclose that OmpR is involved in the survival of *Y. pestis *within macrophages and in building resistance against various environmental perturbations including osmotic stress. DNA microarray and quantitative RT-PCR have been employed to identify a set of OmpR-dependent genes in *Y. pestis. Y. pestis *OmpR simulates *ompC*, *F*, *X*, and *R *directly by occupying the target promoter regions. Noticeably, there is an inducible expression of all of *ompF, C, X*, and *R *at high osmolarity in *Y. pestis*, in contrast to the reciprocal regulation of OmpF and OmpC in *E. coli*. The main difference is that *ompF *expression is not repressed at high osmolarity in *Y. pestis*, which is likely due to the absence of a promoter-distal OmpR-binding site for *ompF*.

## Methods

### Bacterial strains

The wild-type (WT) *Y. pestis *biovar microtus strain 201 is avirulent to humans but highly lethal to mice [22]. The 43 to 666 base pairs of *ompR *(720bp in total length) were replaced by the kanamycin resistance cassette using the one-step inactivation method based on the lambda Red phage recombination system, with the helper plasmid pKD46, to generate the *ompR *mutants of *Y. pestis *(designated as *ΔompR*) [23]. Chromosomal integration of the mutagenic cassette was confirmed by PCR and sequencing using oligonucleotides external to the integrated cassette (data not shown). The elimination of pKD46 in *ΔompR *was verified by PCR.

A PCR-generated DNA fragment containing the *ompR *coding region, together with its promoter-proximal region (~500 bp upstream the coding sequence) and transcriptional terminator (~300 bp downstream), was cloned into the pACYC184 vector harboring a chloramphenicol resistance gene (GenBank accession number X06403), and was then verified by DNA sequencing. The recombinant plasmid was subsequently introduced into *ΔompR*, producing the complemented mutant strain *C-ompR*.

### Bacterial growth and RNA isolation

Overnight cultures (an OD_620 _of about 1.0) of WT or *ΔompR *in the chemically defined TMH medium [24] were diluted 1:20 into the fresh TMH. Bacterial cells were grown at 26°C to the middle exponential growth phase (an OD_620 _of about 1.0). To trigger the high osmolarity conditions in OmpR-related experiments, a final concentration of 0.5 M sorbitol was added, after which the cell cultures were allowed to grow for another 20 min.

Total RNA of bacterial cells was extracted using the TRIzol Reagent (Invitrogen) without the DNA removal step (for RT-PCR and primer extension) or by using MasterPure™RNA Purification kit (Epicenter) with the removal of contaminated DNA (for microarray). Immediately before harvesting, bacterial cultures were mixed with RNAprotect Bacteria Reagent (Qiagen) to minimize RNA degradation. RNA quality was monitored by agarose gel electrophoresis, and RNA quantity was determined using a spectrophotometer.

### Microarray expression analysis

Gene expression profiles were compared between WT and *ΔompR *using a *Y. pestis *whole-genome cDNA microarray as described in a previous work [25]. RNA samples were isolated from four individual bacterial cultures as biological replicates for each strain. The dual-fluorescently (Cy3 or Cy5 dye) labeled cDNA probes, for which the incorporated dye was reversed, were synthesized from the RNA samples. These were then hybridized to 4 separated microarray slides. A ratio of mRNA levels was calculated for each gene. Significant changes of gene expression were identified using the SAM software [26]. After the SAM analysis, only genes with at least two-fold changes in expression were collected for further analysis.

### Real-time RT-PCR

Gene-specific primers were designed to produce a 150 to 200 bp amplicon for each gene (all the primers used in this study were listed in the Additional file 1). The contaminated DNAs in the RNA samples were further removed using the Amibion's DNA-free™Kit. cDNAs were generated using 5 μg of RNA and 3 μg of random hexamer primers. Using 3 independent cultures and RNA preparations, real-time RT-PCR was performed in triplicate as described previously through the LightCycler system (Roche), together with the SYBR Green master mix [23]. On the basis of the standard curves of 16 S rRNA expression, the relative mRNA level was determined by calculating the threshold cycle (ΔCt) of each gene using the classic ΔCt method. Negative controls were performed using 'cDNA' generated without reverse transcriptase as templates. Reactions containing primer pairs without templates were also included as blank controls. The 16 S rRNA gene was used as an internal control to normalize all the other genes. The transcriptional variation between the WT and mutant strains was calculated for each gene. A mean ratio of 2 was taken as the cutoff of statistical significance.

### Primer extension assay

For the primer extension assay [23], about 10 μg of total RNA from each strain was annealed with 1 pmol of [γ-^32^P] end-labeled reverse primer. The extended reverse transcripts were generated as described in the protocol for Primer Extension System-AMV Reverse Transcriptase (Promega). The yield of each primer extension product indicates the mRNA expression level of the corresponding gene in each strain, which can then be used to map the 5' terminus of RNA transcript for each gene. The same labeled primer was also used for sequencing with the fmol^® ^DNA Cycle Sequencing System (Promega). The primer extension products and sequencing materials were concentrated and analyzed by 8 M urea-6% polyacrylamide gel electrophoresis. The result was detected by autoradiography (Kodak film).

### LacZ reporter fusion and β-galactosidase assay

The 500 to 600 bp upstream DNA region of each indicated gene (Table 1) was obtained by PCR with the ExTaq™ DNA polymerase (Takara) using *Y. pestis *201 genome DNA as the template. PCR fragments were then cloned directionally into the *Eco*RI and *Bam*HI sites of plasmid pRW50, which harbors a tetracycline resistance gene and a promoterless *lacZ *reporter gene [27]. Correct cloning was verified through DNA sequencing. *Y. pestis *was then transformed with the recombinant plasmids and grown as described in microarray analysis. The empty plasmid pRW50 was also introduced into both strains as negative control. β-galactosidase activity was measured on cellular extracts using the β-Galactosidase Enzyme Assay System (Promega) [23]. Assays were performed in triplicate. A mean value of fold change was taken as the cutoff of statistical significance.

**Table 1:** Genes tested in both computational and biochemical assays

Gene ID	Gene	Regulation	Computational matching of regulatory consensus			Position of DNA fragment used §	
				
			Position§	Sequence	Score	LacZ	Footprinting
YPO1222	*ompC*	+	D-110...-91	ATAAATACTTGTTGCAATTT	7.06	-379...+130	-245...+31
YPO1411	*ompF*	+	R-99...-80	TTTACATTTTGTAACACATA	11.57	-328...+143	-389...+69
YPO2506	*ompX*	+	R-82...-63	GAAATTCTTTGTTACATGAA	6.03	-374...+123	-191...+89
YPO0136	*ompR*	+	D-81...-62	AATAAGCTTTGTAACAATTT	10.34	-409...+83	-238...+14

§, The numbers indicate the nucleotide positions upstream of the transcription start sites

+, positive and direct regulation

-, negative and direct regulation

### Preparation of His-OmpR protein

The entire coding region of *ompR *was amplified from *Y. pestis *201 and then cloned directionally into the respective *Bam*HI and *Hind*III sites of plasmid pET28a. This was later verified through DNA sequencing. The recombinant plasmid encoding a His-protein was transformed into BL21λDE3 cells. Over-expression of His-OmpR in the LB medium was induced by adding 1 mM isopropyl-b-D-thiogalactoside. The over-expressed protein was purified under native conditions with nickel-loaded HiTrap Chelating Sepharose columns (Amersham). The purified and eluted protein was concentrated to a final concentration of 0.1 to 0.3 mg/ml with the Amicon Ultra-15 (Millipore), which was confirmed by SDS-PAGE for purity. The purified protein was stored at -80°C.

### DNase I footprinting

The promoter DNA regions (Table 1) were prepared by PCR amplification performed with the promoter-specific primer pairs (see Additional file 1 for primer sequences), including a 5'-^32^P-labeled primer (either forward or reverse) and its non-labeled counterpart. The PCR products were purified using QiaQuick cleanup columns (Qiagen).

Increasing amounts of purified His-protein were incubated with the labeled DNA fragment (2 to 5 pmol) for 30 min at room temperature in a binding buffer containing 10 mM Tris-HCl (pH7.4), 50 mM KCl, 0.5 mM DTT, 1 mM MgCl_2_, 4% glycerol, 0.05 mg/ml BSA, 0.05 mg/ml shared salmon sperm DNA and 0.5 mM EDTA, with a final volume of 10 μl. Afterwards, 25 mM of fresh acetyl phosphate was added in the binding buffer and incubated with purified His-OmpR for 30 min to achieve the OmpR phosphorylation, after which the labeled DNA was added for additional incubation for 30 min. Prior to DNA digestion, 10 μl of Ca^2+^/Mg^2+ ^solution (5 mM CaCl_2 _and 10 mM MgCl_2_) was added, followed by incubation for 1 min at room temperature. The optimized RQ1 RNase-Free DNase I (Promega) was then added to the reaction mixture, which was subsequently incubated at room temperature for 30 to 90 s. The cleavage reaction was stopped by adding 9 μl of the stop solution (200 mM NaCl, 30 mM EDTA and 1% SDS) followed by DNA extraction and precipitation. The partially digested DNA samples were then analyzed in a 6% polyacrylamide/8 M urea gel. Protected regions were identified by comparing these with the sequence ladders. For sequencing, the fmol^® ^DNA Cycle Sequencing System (Promega) was used. The result was detected by autoradiography (Kodak film).

### Computational promoter analysis

The 300 bp promoter regions upstream of the start codon of each indicated gene were retrieved with the '*retrieve-seq*' program [28]. The '*matrices-paster' *tool [28] was used to match the relevant position-specific scoring matrix (PSSM) within the above promoter regions.

### Environmental stress experiments

*Y. pestis *strain 201 inoculated into TMH was grown to the early logarithm phase at 26°C. To determine the effect of high osmolarity stress on *Y. pestis*, the log-phase cells were kept incubated at 26°C for 20 min in the presence of 1.5 M sorbitol. For high-temperature stress experiments, log-phase cells were transferred to pre-warmed 50°C tubes and incubated at 50°C for 5 min. For low pH stress experiments, log-phase cells were incubated at 37°C in TMH medium adjusted by adding 2 M HCl to pH 3.0 for 10 min. To test the effect of oxidative stress, the cells were incubated for 10 min in 220 mM H_2_O_2_. The bacterial viable count after exposure to the appropriate stresses was determined by pelleting the appropriate dilutions on the BHI agar plates, which were then incubated at 26°C for 36 h.

### Macrophage infection assay

J774A.1 mouse macrophage cells (6 × 10^5^) were seeded in 24-well tissue culture plates (0.5 ml/well) and maintained in the minimum essential medium (MEM) containing the modified Eagle's medium (Invitrogen) supplemented with 10% heat-inactivated fetal bovine serum, 2 mM L-glutamine until confluence was achieved at 37°C under 5% CO_2_. WT and *ΔompR *were grown in TMH as described above. The cultures were collected and suspended in the MEM medium and then respectively added to cell monolayers in 24-well tissue culture plates at a multiplicity of infection generally of 20:1 (bacteria to macrophages). After incubation at 37°C for 1 h to permit phagocytosis, 6 wells of infected cell monolayers were washed thrice with 1× phosphate-buffered saline (PBS). Afterwards, the number of total macrophage cell-associated bacteria was determined. Cell-associated bacteria were determined by harvesting in 0.5 ml of 0.1% Triton X-100 in 1× PBS. After 10 min, infected cell lysates were collected serially and diluted 10-fold in PBS; on the other hand, viable bacterial CFU was determined as described above. A second set of 6 infected monolayer wells were washed twice with 1× PBS. MEM medium supplemented with 200 μg/ml gentamicin (Invitrogen) was added to these wells for 1 h to kill extracellular bacteria. The infected monolayers were then lysed and treated as described above to determine the number of intracellular bacteria. Each experiment was repeated three or four times on different days, and each bacteria sample was used to infect at least four wells of macrophage monolayers.

## Results

### *Non-polar mutation of *ompR

Given that the coding regions of *ompR *and *envZ *overlap in the *ompB *operon, a partial segment of the coding region of *ompR *was replaced by the kanamycin resistance cassette to generate the *ompR *mutant (*ΔompR*). Real-time RT-PCR was performed to assess the *ompR *mRNA levels in WT, *ΔompR*, and *C-ompR *(the complemented mutant). The *ompR *transcript was lacking in *ΔompR*, while it was restored in *C-ompR *relative to WT (data not shown), indicating successful mutation and complementation. To prove the non-polar mutation of *ompR*, we constructed the pRW50-harboring fusion promoter consisting of a promoter-proximal region of *ompF *and promoterless *lacZ*, and then transformed into WT, *ΔompR *and *C-ompR*, respectively (Additional file 2). The *ompF *gene was positively regulated by OmpR as determined by several distinct methods (see below). As expected, the *ompF *promoter activity (β-galactosidase activity) decreased significantly in *ΔompR *relative to WT grown at high medium osmolarity (0.5 M sorbitol); however, it showed almost no difference between WT and *C-ompR*, thereby confirming that the *ompR *mutation was nonpolar.

### *Phenotypes of *ΔompR

**Figure 1:**
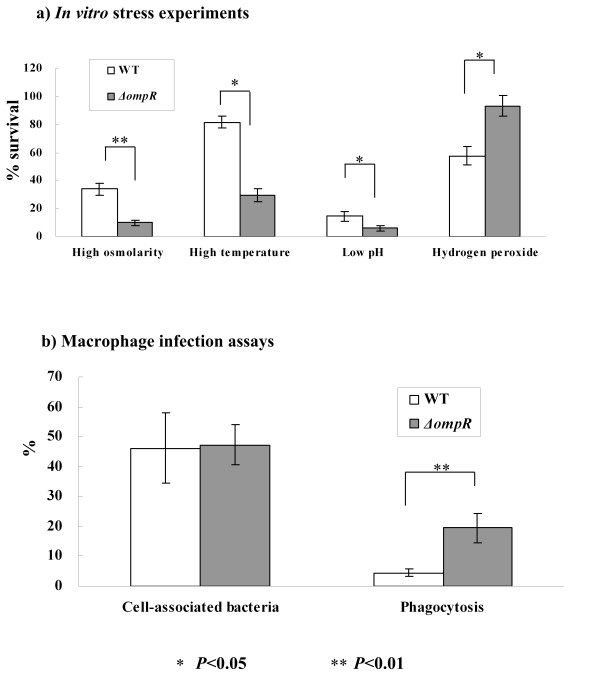
**Phenotypes of *ΔompR***. a) WT or *ΔompR *was characterized for the ability to survive under a range of environmental stresses associated with macrophage-killing mechanisms. The '% survival' values indicate the percentage of viable bacteria after exposure to the environmental stresses. b) WT or *ΔompR *was used to infect macrophages so as to investigate bacterial resistance to phagocytosis *in vivo *and adhesion on the cell surface. The percentage of cell-associated bacteria was determined by dividing the total number of cell-associated bacteria into the total CFU in the inoculum, while the percentage of phagocytosis was calculated by dividing the number of cell-associated bacteria by the number of intracellular bacteria. Finally, student's *t *test was carried out to determine the statistical significance (*P *< 0.05).

The *ΔompR *mutant was characterized for its ability to survive under a range of *in vitro *stress conditions associated with macrophage-killing mechanisms (Figure 1a). In comparison to its WT parent strain, *ΔompR *was significantly more sensitive to high salt, high osmolarity, and high temperature. Both WT and mutant strains were extremely sensitive to acid shock without any significant difference between them; in addition, *ΔompR *seemed more resistant to hydrogen peroxide. Therefore, OmpR should play roles in the regulation of the adaptation to well-documented hyperosmotic stress and additional environmental perturbations, such as heat and oxidative stresses.

Macrophage infection assay was performed to investigate the role of OmpR in the initiation of bacterial strategies against macrophages. A significant increase in the percentage of phagocytosis for *ΔompR *relative to WT (Figure 1b) suggested that the mutant was more susceptible to phagocytosis. For the percentage of cell-associated bacteria, no difference was observed between the WT and mutant strains, thereby suggesting that OmpR does not have a role in the bacterial adhesion to phagocytes (Figure 1b).

### OmpR-dependent genes

By standard cDNA microarray experiments, the mRNA level of each gene was compared between *ΔompR *and WT grown at 0.5 M sorbitol. In all, 224 genes were affected by the *ompR *mutation. These genes represented more than 4% of total protein-encoding capacity of *Y. pestis *and were distributed in 24 functional categories according to the genome annotation of *Y. pestis *CO92 [29], indicating the global regulatory effect of OmpR. The microarray data (GSE26601) had been deposited in Gene Expression Omnibus (GEO).

Known OmpR-binding sites from *S. enterica *and *E. coli *were collected and aligned to generate an OmpR consensus that was a position-specific scoring matrix (PSSM) (Additional file 3), in which each row and column represents a position and a nucleotide, respectively. Given that the OmpR protein sequences were highly conserved among *S. enterica*, *E. coli *and *Y. pestis *(data not shown), this PSSM represents conserved signals for OmpR recognition of promoter DNA regions for all these bacteria. Thus, the PSSM generated from the pre-existing data in *E. coli *and *S. enterica *can be used to predict computationally the presence of OmpR consensus-like elements within a target promoter-proximal sequence of *Y. pestis*.

Accordingly, the 300 bp upstream promoter DNA regions of the 234 mpR-dependent genes that were disclosed by microarray were scanned using PSSM. This computational promoter analysis generated a weight score for each gene, and a higher score denoted the higher probability of OmpR binding. With a cutoff value of 7, only 14 genes gave predicted OmpR consensus-like elements (Additional file 4); these were then subjective to real-time RT-PCR analysis to compare their mRNA levels between *ΔompR *and WT. In accordance with microarray results, RT-PCR disclosed that all 14 genes were expressed differentially in *ΔompR *relative to WT.

In addition to these 14 genes, we still included 2 additional ones, namely, *ompR *and *X*, for further analysis. The OmpR-dependent expression of *ompR *could not be determined by microarray and RT-PCR since the coding region of *ompR *was deleted from the *ΔompR *mutant strain. The *ompX *gene was discarded by SAM in the microarray assay (which could be attributed to the fact that the repeatability of the 8 replicated data points of this gene were unacceptable by SAM), although it gave a more than 2-fold mean change of expression between WT and *ΔompR*. Further biochemical assays (see below) confirmed that OmpR did regulate these genes.

Altogether, we validated 16 genes whose transcriptions were OmpR-dependent (Additional file 4), including *ompR*, *C*, *F*, and *X *that were further characterized below (Table 1). All of these represented the candidates of direct OmpR targets (*ompR*, *C*, *F*, and *X *were confirmed below) since OmpR consensus-like sequences were predicted within their respective promoter-proximal regions.

### *Direct regulation of *ompC, F *and *X *by OmpR*

**Figure 2:**
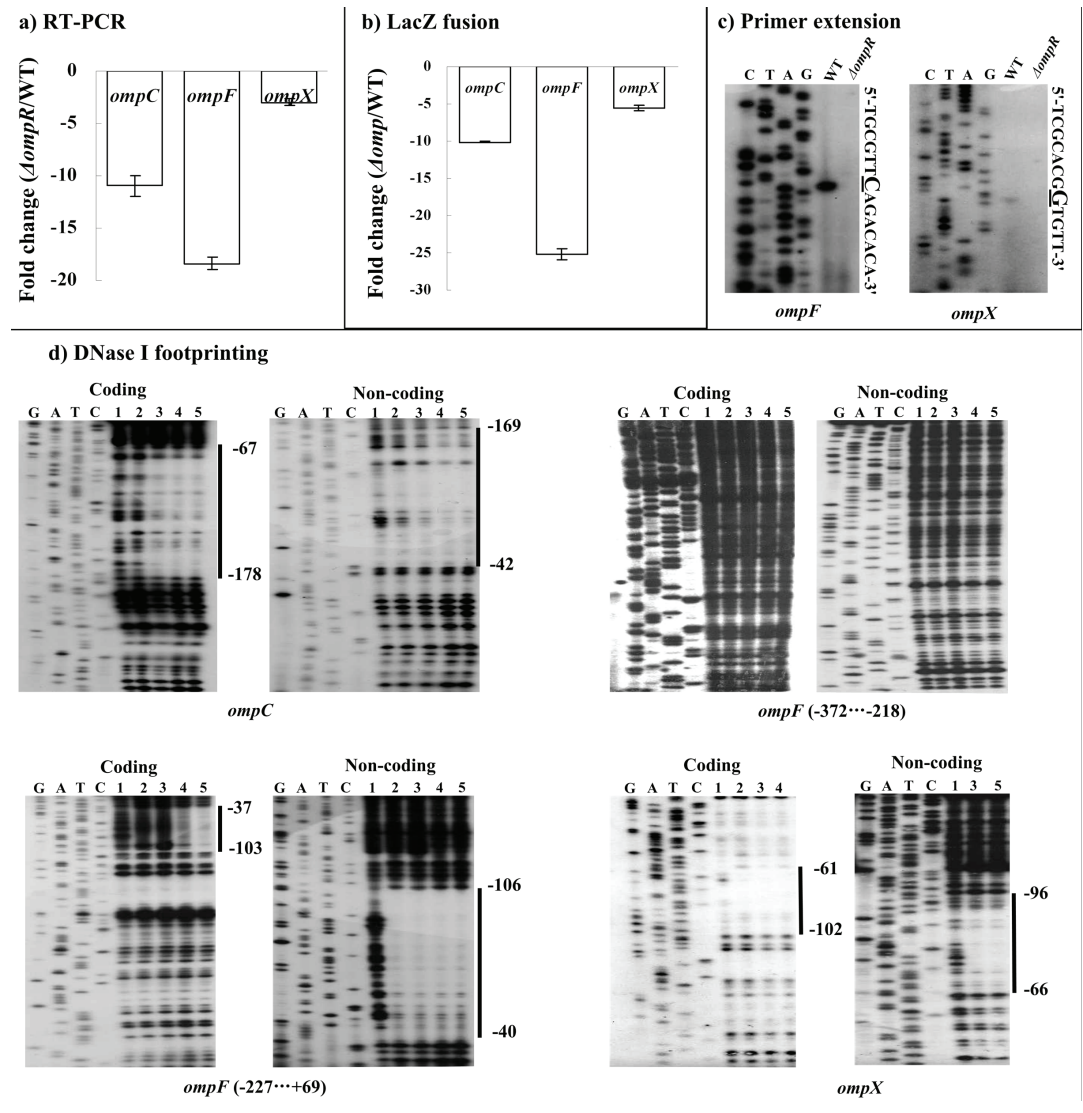
**Regulation of *ompC*, *F *and *X *by OmpR**. a) Real-time RT-PCR. The mRNA levels of each indicated gene were compared between *ΔompR *and WT. This figure shows the increased (positive number) or decreased (minus one) mean fold for each gene in *ΔompR *relative to WT. b) LacZ fusion reporter. A promoter-proximal region of each indicated gene was cloned into pRW50 containing a promoterless *lacZ *reporter gene, and transformed into WT or *ΔompR *to determine the promoter activity (β-galactosidase activity in cellular extracts). The empty plasmid was also introduced into each strain as negative control, which gave extremely low promoter activity (data not shown). Positive and minus numbers indicate the increased and decreased mean folds, respectively, for the detecting promoter activity in *ΔompR *relative to WT. c) Primer extension. Primer extension assays were performed for each indicated gene using total RNAs isolated from the exponential-phase of WT or *ΔompR*. An oligonucleotide primer complementary to the RNA transcript of each gene was designed from a suitable position. The primer extension products were analyzed with 8 M urea-6% acrylamide sequencing gel. Lanes C, T, A, and G represent the Sanger sequencing reactions; on the right side, DNA sequences are shown from the bottom (5') to the top (3'), and the transcription start sites are underlined. d) DNase I footprinting. The labeled DNA probe was incubated with various amounts of purified His-OmpR (lanes 1, 2, 3, 4, and 5 contained 0, 5, 10, 15 and 20 pmol, respectively) with the addition of acetyl phosphate, and subjected to DNase I footprinting assay. Lanes G, A, T, and C represent the Sanger sequencing reactions, and theprotected regions (bold lines) are indicated on the right-hand side. The numbers indicate the nucleotide positions upstream of the transcriptional start sites.

The mRNA levels of each of *ompC*, *F*, and *X *were compared between *ΔompR *and WT at 0.5 M sorbitol using real-time RT-PCR (Figure 2a). The results showed that the mRNA level of *ompC*, *F*, and *X *decreased significantly in *ΔompR *relative to WT. Further *lacZ *fusion reporter assays demonstrated that the promoter activity of *ompC*, *F*, and *X *decreased significantly in *ΔompR *relative to WT, thereby confirming the RT-PCR results. Primer extension experiments were further conducted for *ompC*, *F*, and *X *with *ΔompR *and WT at 0.5 M sorbitol (Figure 2c). A single primer extension product was detected for each of *ompF *and *X*, after which the 5' terminus of RNA transcript (transcription start site) for each gene was identified accordingly. The yield of primer extension product indicated the mRNA expression level for each gene in the corresponding strain. These results further verified the above RT-PCR data for *ompF *and *X*. However, we failed to detect the primer extension product for *ompC *in both *ΔompR *and WT after repeated efforts using different primers. This could be attributed to the failure to synthesize the primer extension product for *ompC *by polymerase.

Given that OmpR consensus-like sequences were found within the promoter regions of *ompC*, *F *and *X *(Table 1), DNase I footprinting experiments (Figure 2d) were subsequently performed with both coding and non-coding strands of the corresponding promoter-proximal DNA fragments. The purified His-OmpR-P protein protected a single distinct region (OmpR-binding site) within each target promoter region in a dose-dependent pattern. Taken together, the OmpR regulator stimulated the expression of *ompC*, *F*, and *X *through the process of OmpR-promoter DNA association.

### Autoregulation of OmpR

**Figure 3:**
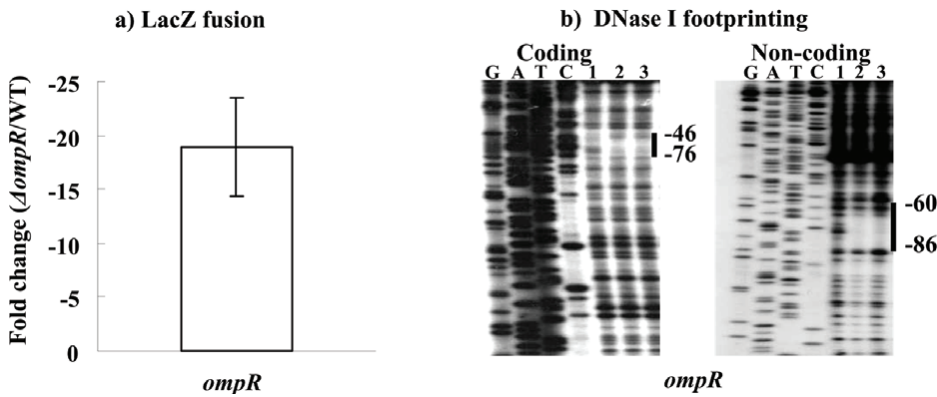
**Autoregulation of OmpR but not CRP**. a) LacZ fusion reporter. A recombinant pRW50 that contained a promoter-proximal region of *ompR *was transformed into WT or *ΔompR *to determine the promoter activity. This figure shows the decreased mean fold for the *ompR *promoter activity in *ΔompR *relative to WT. d) DNase I footprinting. For DNase I digestion, the labeled promoter-proximal region of *ompR *was incubated with various amounts of purified, acetyl phosphate-treated His-OmpR (lanes 1, 2, and 3 contained 0, 10 and 20 pmol, respectively). Lanes G, A, T, and C represent the Sanger sequencing reactions, and the protected regions (bold lines) are indicated on the right-hand side. The numbers indicate the nucleotide positions upstream the transcriptional start sites.

According to the *lacZ *fusion reporter assay (Figure 3a); there was a more than 10-fold decrease of the *ompR *promoter activity in *ΔompR *relative to WT at 0.5 M sorbitol, thereby indicating that OmpR stimulated the promoter activity of its own gene. The subsequent DNase I footprinting experiments (Figure 3a) showed that His-OmpR-P protected a single region within the *ompR *promoter. Therefore, OmpR stimulated its own gene at the transcriptional level, which was mediated through the binding of OmpR-P to its own promoter.

### *Expression of *ompC, F, × *and *R *under different osmotic conditions*

**Figure 4:**
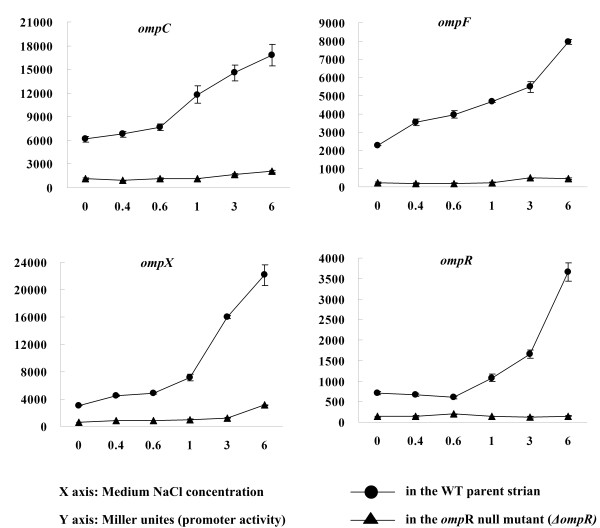
**Promoter activity *ompC*, *F*, *X *and *R *under different concentrations of NaCl**. The *lacZ *fusion reporter plasmid for each of *ompC*, *F*, *X*, and *R *was transformed into WT or *ΔompR *to determine the β-galactosidase activity (miller unites), respectively. Bacterial cultures in the LB broth (0.5% yeast extract, 1% tryptone and 1% NaCl) at the middle exponential growth phase (an OD_620 _of about 1.0) were diluted 1:50 into the fresh LB broth. Bacterial cells were grown at 26°C to an OD_620 _of about 1.0, pelleted and resuspended in the fresh LB broth containing 0, 0.4, 0.6, 1, 3 and 6% NaCl, respectively, and allowed to continue growing at 26°C for 20 min for bacterial harvest.

The promoter activities of *ompC*, *F*, *X*, and *R *were each determined in WT or *ΔompR *grown in the LB broth using *lacZ *fusion reporter assay (Figure 4). The LB broth was used here instead of the TMH medium since it was convenient to modify the medium osmolarity in the LB medium by adding different concentrations of NaCl. The results demonstrated that the promoter activities of *ompC*, *F*, *X*, and *R *were enhanced dramatically with the increasing of NaCl concentration (i.e., medium osmolarity) in WT. However, this effect almost disappeared in the *ΔompR *mutant, suggesting that OmpR mediated the noticeably inducible transcription of these genes upon exposure to hyperosmotic stress.

## Discussion

### Conserved OmpR-dependent phenotypes among pathogenic yersiniae

As shown in *Y. enterocolitica *[30,31], *Y. pseudotuberculosis *[32] and *Y. pestis *(the present work) in a conserved manner, OmpR is involved in the resistance to phagocytosis and/or survival within macrophages and controls the adaptation to various killing mechanisms used by macrophages against pathogens. The *ompR *mutants of both *Y. enterocolitica *[30] and *Y. pseudotuberculosis *[32] are attenuated in the mouse model. OmpR is a repressor of the *inv *gene, which encodes the major virulence determinant invasin in *Y. enterocolitica *[33]. In *Y. pseudotuberculosis*, OmpR regulates positively the urease expression to enhance acid survival [34], whereas it controls negatively the expression of FlhD and FlhC that form a heterohexameric transcriptional activator of the flagellar genes [35]. In this work, the *ompR *mutation likely had not affect on the virulence of *Y. pestis *201, which was a human-attenuated enzootic strain in a mouse model after subcutaneous infection (data not shown). In this light, a further animal virulence test using a typical epidemic strain is hereby required.

### *Global regulatory effect of OmpR in *Y. pestis

The microarray expression analysis disclosed a set of 224 genes that were affected by the *ompR *mutation in *Y. pestis*. A similar global regulatory effect of OmpR has been observed in *E. coli *[36]. Real-time RT-PCR or *lacZ *fusion reporter assay further validated 16 OmpR-dependent genes, for which OmpR consensus-like sequences were found within their promoter regions. These 16 genes represent the candidates of direct OmpR targets in *Y. pestis*, of which *ompR*, *C*, *F*, and *X *were further characterized for the molecular mechanisms of regulation by OmpR.

### Transcriptional auto-stimulation of OmpR

We confirmed the direct transcriptional auto-stimulation of *ompR *in *Y. pestis*. In addition, the *ompR *promoter activity was dramatically and persistently enhanced in *Y. pestis *with the increasing medium osmolarity, which was mediated by OmpR itself.

The auto-stimulation of the *ompB *operon appears to be conserved in *Y. pestis*, *E. coli*, and *S. enterica *[3]. The histone-like protein HN-S is a negative regulator of *ompB *expression in both *E. coli *[37] and *S. enterica*, and the role of OmpR-P in autoinduction is to help to counteract repression by H-NS [3]. In conclusion, transcription from the *ompB *promoter is repressed by H-NS and requires OmpR-P for induction; in addition, EnvZ (as a sensor kinase) and acetyl phosphate collaborate to produce the optimum level of OmpR-P needed for autoinduction [3,37].

### Osmotic regulation of porins

Previous works [38,39] have proposed that the shift in cellular porin levels reflects the adaptation of enteric bacteria to a transition between a life in the mammalian gut as 'high osmolarity' and a free-living aqueous state as 'low osmolarity.' OmpC expression is favored in the gut, while OmpF is predominately expressed in the aqueous habitats. Compared to OmpF, OmpC has smaller pore and, hence, slower flux [39]. The smaller pore size of OmpC can aid in excluding harmful molecules, such as bile salts, in the gut. In the external aqueous environment, the larger pore size of OmpF can assist in scavenging for scarce nutrients.

The amounts of OmpC and OmpF in the outer membrane of *E. coli *vary depending on the medium osmolarity, and their relative levels fluctuate in a reciprocal manner; in addition, the *ompX *expression is inducible upon early exposure to high osmolarity, which is accompanied by the repressed expression of OmpF and OmpC [12,14]. In this work, *ompX*, *C*, and *F *were up-regulated dramatically upon the increase of medium osmolarity in *Y. pestis*. This is in stark contrast to the classic reciprocal regulation of these same proteins. OmpF is over-expressed at low osmolarity in *E. coli*, while it is likely no longer employed by *Y. pestis*. How *Y. pestis *express porins during the transition from mammalian blood or lymph into the flea gut remains unclear. Nevertheless, we could postulate that *Y. pestis *has lost the mechanism of over-expressing the relevant porin at low osmolarity, since it always encounters high osmolarity environments in its life in mammalian blood or lymph and flea midgut, and has a rare chance of living in the environment [40]. Another issue involves whether or not the mechanism of porin regulation observed is specific for *Y. pestis*, or conserved in *Y. pseudotuberculosis *with a life transitioning from free-living environments into mammalian gut (e.g., *E. coli *and *S. enterica*). A comparison between porin regulation in *Y. pestis *and *Y. pseudotuberculosis *may provide first insights into possible evolutionary forces selecting for altered gene regulation.

OmpC is highly expressed in *S. typhi *independent of medium osmolarity, whereas OmpF is osmoregulated as it is in *E. coli *[41]. In addition, OmpC is always more abundant than OmpF in *S. typhi*, regardless of the growth conditions [42]. The lack of osmoregulation of OmpC expression in *S. typhi *is determined in part by the ompB operon, as well as by other unknown trans-acting regulators in *S. typhi *[42]. The evidenced differences in porin regulation (as seen in *Y. pestis*, *S. typhi*, and *E. coli*) could possibly have an effect on how these bacteria survive in the environment or during pathogenesis.

### Organization of OmpR-recognized promoter regions

**Figure 5:**
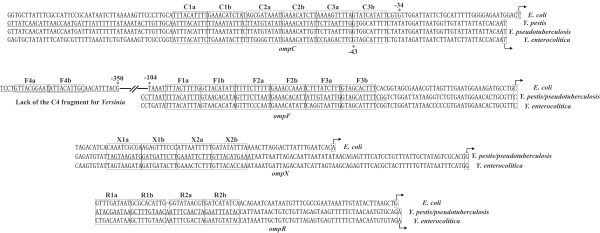
**OmpR consensus-like sequences within the target promoter regions**. The underlined segments are OmpR binding sites determined by DNase I footprinting in *Y. pestis*. The boxed areas represent the sub-elements of OmpR consensus-like sequence. This figure also shows the three (e.g., C1-C2-C3) or 2 (e.g., X1-X2) tandems of OmpR consensus-like sequences, where each 20 bp tandem has been divided into two 10 bp sub-elements (boxed).

The present study confirmed that OmpR-P recognized the promoter regions of *ompC*, *F*, *X*, and *R *to regulate the target promoter activity. We aligned OmpR-binding sites within relevant promoter regions from *E. coli *and the 3 pathogenic yersiniae (Figure 5). Then, 3 tandems of OmpR consensus-like sequences were detected for *ompC *(C1-C2-C3) or *ompF *(F1-F2-F3), while 2 tandems were detected for *ompR *(R1-R2) or *ompX *(X1-X2) in yersiniae. As expected, each OmpR consensus-like element consisted of 20 base pairs that can be divided into two 10 bp sub-elements (e.g., X1a and X1b), providing a tandem binding site for 2 OmpR-P molecules [43]. These results confirmed that multiple OmpR proteins occupied the target promoter in a tandem manner to regulate its activity.

Remarkably, F1-F2-F3 and C1-C2-C3 were detected for *ompF *and *ompC*, respectively, although F4 was absent for *ompF*. Given that OmpR-P binding to the promoter-distal F4 site at high osmolarity likely formed a loop that interacted with OmpR-P molecules binding to the promoter-proximal F1, F2, and F3 sites--thereby blocking the transcription of *ompF*--the absence of F4 in *Y. pestis *destroyed the above blocking mechanism. Indeed, *ompF *was up-regulated gradually in an OmpR-dependent manner upon the increase of medium osmolarity in *Y. pestis*.

### *Regulation of *ompX *by OmpR*

OmpR still recognized the *ompX *promoter region and stimulated its transcription in *Y. pestis*. To our knowledge, this is the first report of *ompX *regulation by OmpR, although OmpR consensus-like sequences have also been found within the *ompX *upstream region in *E. coli *(data not shown) and *E. aerogenes *[6]. At the very least, the direct transcriptional regulation of *ompX *by OmpR is conserved in the above-mentioned bacteria.

## Conclusion

The *ompR *mutation in *Y. pestis *strain 201 attenuated the resistance to phagocytosis as well as the adaptation to various stressful conditions met in macrophages; however, it had no effect on the virulence of this pathogen. Microarray expression analysis disclosed at least 232 genes whose transcription was affected by the OmpR-dependent in *Y. pestis*. Real-time RT-PCR or *lacZ *fusion reporter assays were then conducted to validate 16 OmpR-dependent genes, including *ompC*, *F*, *X*, and *R*. Notably, OmpR consensus-like sequences were found within the upstream DNA regions of these 16 genes, thereby representing the candidates of direct OmpR targets. *ompC*, *F*, *X*, and *R *were subsequently proven to be directly regulated by OmpR through OmpR-promoter DNA association. All of *ompC*, *F*, *X*, and *R *were up-regulated dramatically with the increase in medium osmolarity, which was mediated by OmpR that occupied the target promoter regions in a tandem manner. The inducible expressions of the pore-forming proteins OmpF, C, and × at high osmolarity in *Y. pestis *were in contrast to their reciprocal regulations in *E. coli*. The main difference was that *ompF *expression was not repressed at high osmolarity in *Y. pestis*, which was likely due to the absence of a promoter-distal OmpR-binding site for *ompF*.

## Authors' contributions

DZ and RY conceived the study and designed the experiments. HG and YZ performed all the experiments. YH contributed to phenotypic experiments. YH and DZ performed the microarray experiments. LY, XL, and ZG contributed to RT-PCR, primer extension assay, and DNA binding assays. ZG and YT participated in protein expression and purification. HG and DZ performed computational analysis and figure construction. The manuscript was written by HG and DZ, and revised by RY. All the authors read and approved the final manuscript.

## Supplementary Material

Additional file 1 **Oligonucleotide primers used in this study**. Click here for file

Additional file 2 **Promoter activity *ompF *within WT, *ΔompR *and *C-ompR***. Click here for file

Additional file 3 **Construction of the OmpR consensus (PSSM)**. Click here for file

Additional file 4 **The 16 verified OmpR-dependent genes**. Click here for file

## References

[B1] Pratt LA Hsing W Gibson KE Silhavy TJ From acids to osmZ: multiple factors influence synthesis of the OmpF and OmpC porins in Escherichia coli Mol Microbiol 1996 20 5 911 917 10.1111/j.1365-2958.1996.tb02532.x 8809744

[B2] Feng X Oropeza R Walthers D Kenney LJ OmpR phosphorylation and its role in signaling and pathogenesis ASM News 2003 69 390 395

[B3] Bang IS Audia JP Park YK Foster JW Autoinduction of the ompR response regulator by acid shock and control of the Salmonella enterica acid tolerance response Mol Microbiol 2002 44 5 1235 1250 10.1046/j.1365-2958.2002.02937.x 12068808

[B4] Basle A Rummel G Storici P Rosenbusch JP Schirmer T Crystal structure of osmoporin OmpC from E. coli at 2.0 A J Mol Biol 2006 362 5 933 942 10.1016/j.jmb.2006.08.002 16949612

[B5] Yamashita E Zhalnina MV Zakharov SD Sharma O Cramer WA Crystal structures of the OmpF porin: function in a colicin translocon Embo J 2008 27 15 2171 2180 10.1038/emboj.2008.137 18636093 PMC2516885

[B6] Dupont M De E Chollet R Chevalier J Pages JM Enterobacter aerogenes OmpX, a cation-selective channel mar- and osmo-regulated FEBS Lett 2004 569 1-3 27 30 10.1016/j.febslet.2004.05.047 15225603

[B7] Guzev KV Isaeva MP Novikova OD Solov'eva TF Rasskazov VA Molecular characteristics of OmpF-like porins from pathogenic Yersinia Biochemistry (Mosc) 2005 70 10 1104 1110 10.1007/s10541-005-0231-z 16271025

[B8] Vogt J Schulz GE The structure of the outer membrane protein OmpX from Escherichia coli reveals possible mechanisms of virulence Structure 1999 7 10 1301 1309 10.1016/S0969-2126(00)80063-5 10545325

[B9] Stoorvogel J van Bussel MJ Tommassen J van de Klundert JA Molecular characterization of an Enterobacter cloacae outer membrane protein (OmpX) J Bacteriol 1991 173 1 156 160 1987115 10.1128/jb.173.1.156-160.1991 PMC207169

[B10] Stoorvogel J van Bussel MJ van de Klundert JA Biological characterization of an Enterobacter cloacae outer membrane protein (OmpX) J Bacteriol 1991 173 1 161 167 1702778 10.1128/jb.173.1.161-167.1991 PMC207170

[B11] Arnold T Poynor M Nussberger S Lupas AN Linke D Gene duplication of the eight-stranded beta-barrel OmpX produces a functional pore: a scenario for the evolution of transmembrane beta-barrels J Mol Biol 2007 366 4 1174 1184 10.1016/j.jmb.2006.12.029 17217961

[B12] Dupont M James CE Chevalier J Pages JM An early response to environmental stress involves regulation of OmpX and OmpF, two enterobacterial outer membrane pore-forming proteins Antimicrob Agents Chemother 2007 51 9 3190 3198 10.1128/AAC.01481-06 17606680 PMC2043185

[B13] Fernandez C Hilty C Bonjour S Adeishvili K Pervushin K Wuthrich K Solution NMR studies of the integral membrane proteins OmpX and OmpA from Escherichia coli FEBS Lett 2001 504 3 173 178 10.1016/S0014-5793(01)02742-9 11532450

[B14] Kawaji H Mizuno T Mizushima S Influence of molecular size and osmolarity of sugars and dextrans on the synthesis of outer membrane proteins O-8 and O-9 of Escherichia coli K-12 J Bacteriol 1979 140 3 843 847 391802 10.1128/jb.140.3.843-847.1979 PMC216723

[B15] Bornet C Saint N Fetnaci L Dupont M Davin-Regli A Bollet C Pages JM Omp35, a new Enterobacter aerogenes porin involved in selective susceptibility to cephalosporins Antimicrob Agents Chemother 2004 48 6 2153 2158 10.1128/AAC.48.6.2153-2158.2004 15155215 PMC415628

[B16] Scott NW Harwood CR Studies on the influence of the cyclic AMP system on major outer membrane proteins of Escherichia coli K12 FEMS Microbiol Lett 1980 9 95 98 10.1111/j.1574-6968.1980.tb05614.x

[B17] Huang L Tsui P Freundlich M Positive and negative control of ompB transcription in Escherichia coli by cyclic AMP and the cyclic AMP receptor protein J Bacteriol 1992 174 3 664 670 1310090 10.1128/jb.174.3.664-670.1992 PMC206141

[B18] Zhou D Yang R Molecular Darwinian evolution of virulence in Yersinia pestis Infect Immun 2009 77 6 2242 2250 10.1128/IAI.01477-08 19289506 PMC2687345

[B19] Brzostek K Raczkowska A The YompC protein of Yersinia enterocolitica: molecular and physiological characterization Folia Microbiol (Praha) 2007 52 1 73 80 10.1007/BF02932142 17571800

[B20] Delihas N Annotation and evolutionary relationships of a small regulatory RNA gene micF and its target ompF in Yersinia species BMC Microbiol 2003 3 1 13 10.1186/1471-2180-3-13 12834539 PMC166144

[B21] Brzostek K Hrebenda J Benz R Boos W The OmpC protein of Yersinia enterocolitica: purification and properties Res Microbiol 1989 140 9 599 614 10.1016/0923-2508(89)90192-7 2626594

[B22] Zhou D Tong Z Song Y Han Y Pei D Pang X Zhai J Li M Cui B Qi Z Genetics of metabolic variations between Yersinia pestis biovars and the proposal of a new biovar, microtus J Bacteriol 2004 186 15 5147 5152 10.1128/JB.186.15.5147-5152.2004 15262951 PMC451627

[B23] Zhan L Han Y Yang L Geng J Li Y Gao H Guo Z Fan W Li G Zhang L The cyclic AMP receptor protein, CRP, is required for both virulence and expression of the minimal CRP regulon in Yersinia pestis biovar microtus Infect Immun 2008 76 11 5028 5037 10.1128/IAI.00370-08 18710863 PMC2573370

[B24] Straley SC Bowmer WS Virulence genes regulated at the transcriptional level by Ca2+ in Yersinia pestis include structural genes for outer membrane proteins Infect Immun 1986 51 2 445 454 3002984 10.1128/iai.51.2.445-454.1986 PMC262351

[B25] Zhou D Qin L Han Y Qiu J Chen Z Li B Song Y Wang J Guo Z Zhai J Global analysis of iron assimilation and fur regulation in Yersinia pestis FEMS Microbiol Lett 2006 258 1 9 17 10.1111/j.1574-6968.2006.00208.x 16630248

[B26] Tusher VG Tibshirani R Chu G Significance analysis of microarrays applied to the ionizing radiation response Proc Natl Acad Sci USA 2001 98 9 5116 5121 10.1073/pnas.091062498 11309499 PMC33173

[B27] El-Robh MS Busby SJ The Escherichia coli cAMP receptor protein bound at a single target can activate transcription initiation at divergent promoters: a systematic study that exploits new promoter probe plasmids Biochem J 2002 368 Pt 3 835 843 10.1042/BJ20021003 12350222 PMC1223047

[B28] van Helden J Regulatory sequence analysis tools Nucleic Acids Res 2003 31 13 3593 3596 10.1093/nar/gkg567 12824373 PMC168973

[B29] Parkhill J Wren BW Thomson NR Titball RW Holden MT Prentice MB Sebaihia M James KD Churcher C Mungall KL Genome sequence of Yersinia pestis, the causative agent of plague Nature 2001 413 6855 523 527 10.1038/35097083 11586360

[B30] Dorrell N Li SR Everest PH Dougan G Wren BW Construction and characterisation of a Yersinia enterocolitica O:8 ompR mutant FEMS Microbiol Lett 1998 165 1 145 151 10.1111/j.1574-6968.1998.tb13139.x 9711851

[B31] Brzostek K Raczkowska A Zasada A The osmotic regulator OmpR is involved in the response of Yersinia enterocolitica O:9 to environmental stresses and survival within macrophages FEMS Microbiol Lett 2003 228 2 265 271 10.1016/S0378-1097(03)00779-1 14638433

[B32] Flamez C Ricard I Arafah S Simonet M Marceau M Phenotypic analysis of Yersinia pseudotuberculosis 32777 response regulator mutants: new insights into two-component system regulon plasticity in bacteria Int J Med Microbiol 2008 298 3-4 193 207 10.1016/j.ijmm.2007.05.005 17765656

[B33] Brzostek K Brzostkowska M Bukowska I Karwicka E Raczkowska A OmpR negatively regulates expression of invasin in Yersinia enterocolitica Microbiology 2007 153 Pt 8 2416 2425 10.1099/mic.0.2006/003202-0 17660406

[B34] Hu Y Lu P Wang Y Ding L Atkinson S Chen S OmpR positively regulates urease expression to enhance acid survival of Yersinia pseudotuberculosis Microbiology 2009 155 Pt 8 2522 2531 10.1099/mic.0.028381-0 19443542

[B35] Hu Y Wang Y Ding L Lu P Atkinson S Chen S Positive regulation of flhDC expression by OmpR in Yersinia pseudotuberculosis Microbiology 2009 155 Pt 11 3622 3631 10.1099/mic.0.030908-0 19643764

[B36] Oshima T Aiba H Masuda Y Kanaya S Sugiura M Wanner BL Mori H Mizuno T Transcriptome analysis of all two-component regulatory system mutants of Escherichia coli K-12 Mol Microbiol 2002 46 1 281 291 10.1046/j.1365-2958.2002.03170.x 12366850

[B37] Tsuzuki M Aiba H Mizuno T Gene activation by the Escherichia coli positive regulator, OmpR. Phosphorylation-independent mechanism of activation by an OmpR mutant J Mol Biol 1994 242 5 607 613 10.1006/jmbi.1994.1610 7932717

[B38] Dorman CJ Chatfield S Higgins CF Hayward C Dougan G Characterization of porin and ompR mutants of a virulent strain of Salmonella typhimurium: ompR mutants are attenuated in vivo Infect Immun 1989 57 7 2136 2140 2543631 10.1128/iai.57.7.2136-2140.1989 PMC313852

[B39] Nikaido H Molecular basis of bacterial outer membrane permeability revisited Microbiol Mol Biol Rev 2003 67 4 593 656 10.1128/MMBR.67.4.593-656.2003 14665678 PMC309051

[B40] Ayyadurai S Houhamdi L Lepidi H Nappez C Raoult D Drancourt M Long-term persistence of virulent Yersinia pestis in soil Microbiology 2008 154 Pt 9 2865 2871 10.1099/mic.0.2007/016154-0 18757820

[B41] Puente JL Verdugo-Rodriguez A Calva E Expression of Salmonella typhi and Escherichia coli OmpC is influenced differently by medium osmolarity; dependence on Escherichia coli OmpR Mol Microbiol 1991 5 5 1205 1210 10.1111/j.1365-2958.1991.tb01894.x 1956297

[B42] Martinez-Flores I Cano R Bustamante VH Calva E Puente JL The ompB operon partially determines differential expression of OmpC in Salmonella typhi and Escherichia coli J Bacteriol 1999 181 2 556 562 9882670 10.1128/jb.181.2.556-562.1999 PMC93410

[B43] Yoshida T Qin L Egger LA Inouye M Transcription regulation of ompF and ompC by a single transcription factor, OmpR J Biol Chem 2006 281 25 17114 17123 10.1074/jbc.M602112200 16618701

