# Supplementary material for: Phenotypic and transcriptional analysis of the osmotic regulator OmpR in Yersinia pestis
Source: BMC Microbiol. 2011 Feb 23;11:39. doi: 10.1186/1471-2180-11-39 (PMC3050692; doi:10.1186/1471-2180-11-39)
Supplement: Additional file 1 — Oligonucleotide primers used in this study. [file 1471-2180-11-39-S1.DOC]

**Oligonucleotide primers used in this study**

| **Gene** | **Primers (5'-3', F/R)** |
| --- | --- |
| **For RT-PCR** | |
| YPO0509 | CAGGATGTTCAGGATGCTATCG/GTTGTCCAAGGTCATTCCAGAG |
| YPO0608 | ATTGTCCTCGCCGCTATATTAC/CACCAACACCGTCACGATAG |
| YPO0609 | CCGCCGAGTTACTGGAAATC/CTTGGCATCTAGGTTGGACAG |
| YPO1222 | CAGCGATGGCGAGTTCTC/AATCACCGAAACGAGTCAGG |
| YPO1411 | CAAGACGGCAACGCAACC/GCCAGCCATGAAGTTGTCAG |
| YPO1634 | TTGTTGCGTCACCATCTG/GGCTTAACCCGTCTTCAC |
| YPO1683 | CAGGAGTGAGTTATTGGGATGG/CGACATTGACTGGCGTAATATC |
| YPO2155 | TCTATCCCGTACCTGTTGTCTC/CATCTGCTGATCGTCACTGTC |
| YPO3034 | AATCCTACCGCCGTTAGCC/TCAGCAATAGCATGGACACAAG |
| YPO3512 | CTGATGATTGCTAACGGTGAAG/ACGATGACCTTGCTGCTTAC |
| YPO3707 | AGGCGTGCGTGAAGAGAC/TCAGGCGAGAAATCATCAAGAC |
| YPO3708 | AGCTGAATCCGTCGGTAATATC/ACCAGAGGCTAATGCGTTATAC |
| YPO4018 | CGTGGCATAGTTGTGGTGAAG/CCATGTCGCTGTGAATAGATCG |
| YPO2506 | TTATGGTCTGGTCGGTGTGG/TTACGGATACGGCTCTGCTC |
| YPO1205 | CCGATATGCTGCCTGAGTTC/CCGTTATTCGTCTCGCCTTC |
| YPO4020 | CACCACGATTATCTCCCGATG/CTAACGCACTGAGGATGACG |
| *ompR* | CAAGATTCTGGTCGTTGATG/ATCAGGTCAAGTACCATCAG |
| *crp* | CTCTCGAATGGTTCCTGTC/ATCATCTCTTTGCCTTCCTC |
| **For gene mutation** | |
| *ompR* | ATGCAAGAGAATCACAAGATTCTGGTCGTTGATGACGATATGTTGTGTCTCAAAATCTCTG/  CCGTCCGGTACAAAGACGTAGCCTAGACCCCACACCGTCTGAAAGCCGCCGTCCCGTCAAG |
| **For protein expression** | |
| *ompR* | GCGGGATCCATGCAAGAGAATCACAAG/GCGAAGCTTTCATGCTTTATTGCCGTCCGG |
| **For LacZ fusion** | |
| *ompR* | GCGGAATTCGAAGTGCTGAAAATTGTTGACC/GCGGGATCCACGTAGACGCATATCGTCATC |
| *ompC* | GCGGAATTCTTGAAGTATGACGGGTATAACG/GCGGGATCCCACTGCCTGCAACCAATAAG |
| *ompF* | GCGGAATTCTACGGCACTGTACCGCATTC/GCGGGATCCGGCTAACAGAGCTGGGATTAC |
| *ompX* | GCGGAATTCGAGACAACGACATCCTTTGGAG/GCGGGATCCCTTTGACCTGCAAAGGCAGA |
| **For DNase I footprinting: genes regulated by OmpR** | |
| *ompC* | CGTGGCTATTATATATCCCTG/TCGAGAATATACTGCCTACG |
| *ompF-1* | GCGTTTAGCCGAAATGGG/CTCGGTGTGCGTGGGAAC |
| *ompF-2* | CACACCGAGAAATGCCAG/GGACACCTGCCACTGC |
| *ompX* | TTGAGCATAATGGTTCGG/CTAACACACACGCTGCTACC |
| *ompR* | GCATTGATAGGGAAACAAGTCG/CGTATTGCCTGGATCTGC |
| **For DNase I footprinting: genes regulated by** CRP | |
| *ompC* | GTGTTAGCCACAATGATAGG/CCATATAGAAAATGCCACC |
| *ompF* | CGTTCCCACGCACACC/GCAGTGTTCCATTCACAGACC |
| *ompX* | CCAGTGTAAATGAAATGCC/ACACCGTGCGACTATAGC |
| *ompR* | GAAGTGCTGAAAATTGTTGACC/ACGTAGACGCATATCGTCATC |
| **For primer extension** | |
| *ompF* | GGCTAACAGAGCTGGGATTAC |
| *ompX* | CTAACACACACGCTGCTACC |
| *ompR* | ACGTAGACGCATATCGTCATC |
