# Supplementary material for: Phenotypic and transcriptional analysis of the osmotic regulator OmpR in Yersinia pestis
Source: BMC Microbiol. 2011 Feb 23;11:39. doi: 10.1186/1471-2180-11-39 (PMC3050692; doi:10.1186/1471-2180-11-39)
Supplement: Additional file 2 — Promoter activity ompF within WT, ΔompR and C-ompR. [file 1471-2180-11-39-S2.DOC]

**
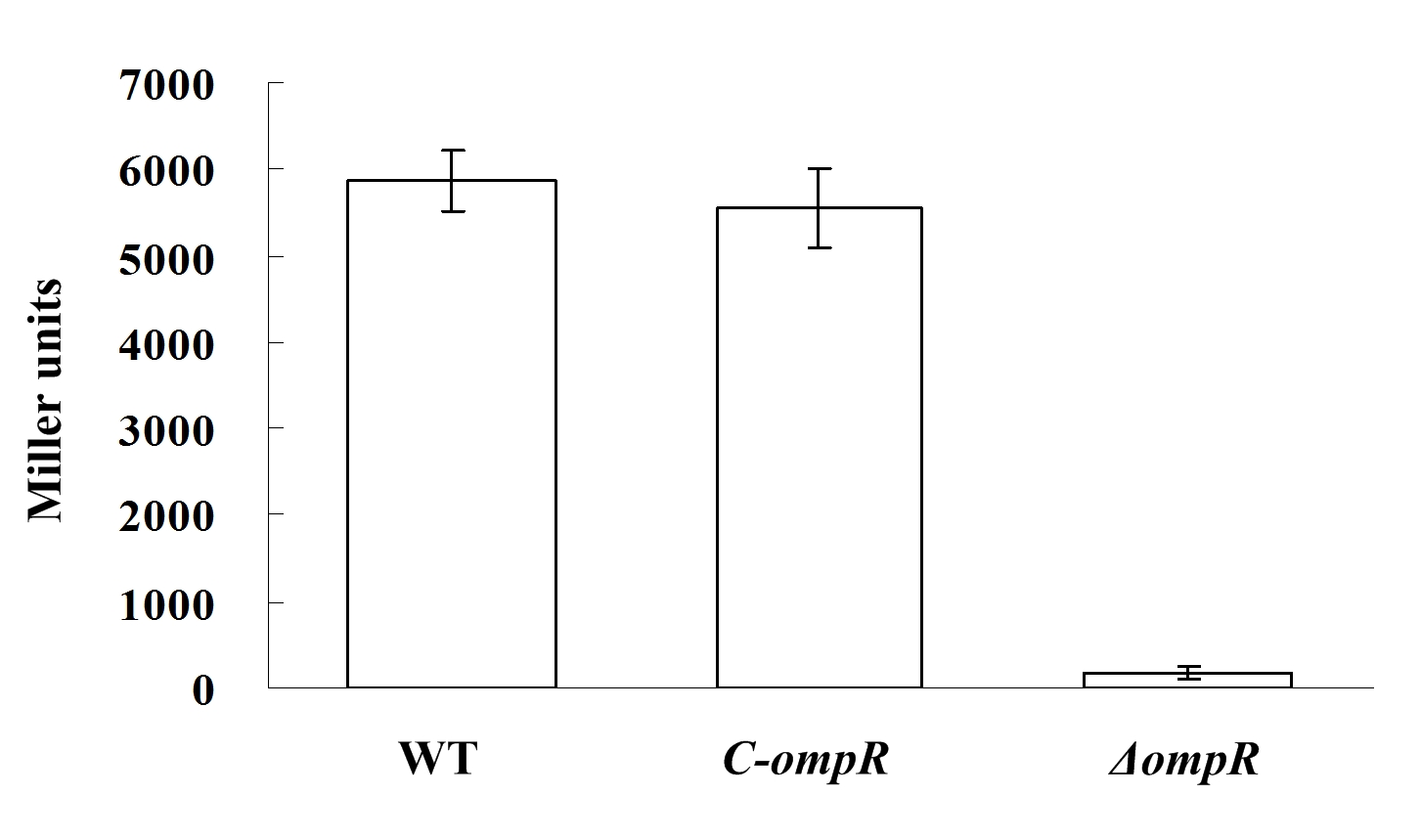
**

A promoter-proximate region of *ompF* was cloned into pRW50 containing a promotorless *lacZ* reporter gene, and transformed into WT, *ΔompR* (the *ompR* null mutant) and *C-ompR* (the complemented mutant) grown at 0.5M sorbitol, respectively, to determine the promoter activity (β-Galactosidase activity in cellular extracts). The empty plasmid was also introduced into each strain as negative control, which gave extremely low promoter activity (data not shown).

Shown in the figure was the mean β-Galactosidase activity (Miller units) in WT, *ΔompR* and *C-ompR*, respectively, subtracted by negative control. The *ompF* gene was positively regulated by OmpR as determined by several distinct methods (see text). As expected herein, the om*pF* promoter activity decreased significantly in *ΔompR* relative to both WT and *C-ompR* (*P*<0.01), but gave no difference between WT and *C-ompR* (*P*>0.05), which confirmed that the *ompR* mutation was nonpolar.
