# Supplementary material for: Phenotypic and transcriptional analysis of the osmotic regulator OmpR in Yersinia pestis
Source: BMC Microbiol. 2011 Feb 23;11:39. doi: 10.1186/1471-2180-11-39 (PMC3050692; doi:10.1186/1471-2180-11-39)
Supplement: Additional file 3 — Construction of the OmpR consensus (PSSM). [file 1471-2180-11-39-S3.DOC]

**Construction of the OmpR consensus**

**(Position-specific scoring matrix)**

OmpR-binding sites in *Salmonella enterica* and *Escherichia coli* were collected and aligned to generate the PSSM by using the *matrices-consensus* tool (12).

**Alignment of OmpR box-like sequences in *Escherichia coli and Salmonella enterica***

| **Bacterium** | **Gene** | **OmpR box-like sequence** | **Reference** |
| --- | --- | --- | --- |
| *E. coli* | *bolA* | TTTAAAGATTGAAACACAGG | (13) |
|  | *csgD* | GTTACATTTAGTTACATGTT | (9) |
|  | *fadL* | GTTACAGCACGTAACATAGT | (8) |
|  | *micF* | AAAAGTTTTAGTATCATATT | (10) |
|  | *micF* | AGCGATAAATGAAACATCTT | (10) |
|  | *micF* | TTTACATTTTGAAACATCTA | (10) |
|  | *nmpC* | GAAACCAAAACTTACATCTT | (2) |
|  | *ompC* | AAAAGTTTTAGTATCATATT | (14) |
|  | *ompC* | AGCGATAAATGAAACATCTT | (14) |
|  | *ompC* | TTTACATTTTGAAACATCTA | (14) |
|  | *ompF* | CTTTATCTTTGTAGCACTTT | (14) |
|  | *ompF* | GTTACGGAATATTACATTGC | (14) |
|  | *ompF* | TTTACTTTTGGTTACATATT | (14) |
|  | *ompF* | TTTTCTTTTTGAAACCAAAT | (14) |
|  | *omrA* | TACACACCTCGTTGCATTTC | (7) |
|  | *omrB* | CAAACCTTTGGTTACACTTT | (7) |
|  | *tppB* | GTAACAGATTATTACAAAGG | (6) |
| *S. enterica* | *csgD* | TTTACATTTGGTTACAAGTT | (5) |
|  | *ompR* | AATATGCTTTGTAACAATTT | (1) |
|  | *ompS1* | AAATATCTTTGTTACATGTT | (11) |
|  | *ompS1* | GAAACAAATTGAAATATTTT | (11) |
|  | *ompS2* | GTTTCCGATAGTAACTTTTG | (4) |
|  | *SsrA* | TAAAAACATCGTAACAGTTT | (3) |
|  | *SsrA* | AGTACATATAGTTTCATCAG | (3) |

**The position-specific scoring matrix for OmpR**

A | 7 9 8 18 6 11 4 10 5 6 2 7 14 19 0 22 4 7 2 2

C | 2 0 3 0 15 3 5 2 0 3 1 0 0 0 23 1 3 6 0 2

G | 7 3 0 2 2 2 5 0 0 3 21 0 0 2 0 0 1 3 4 4

T | 8 12 13 4 1 8 10 12 19 12 0 17 10 3 1 1 16 8 18 16

**References**

1. **Bang, I. S., J. P. Audia, Y. K. Park, and J. W. Foster.** 2002. Autoinduction of the ompR response regulator by acid shock and control of the Salmonella enterica acid tolerance response. Mol Microbiol **44:**1235-50.

2. **Castillo-Keller, M., P. Vuong, and R. Misra.** 2006. Novel mechanism of Escherichia coli porin regulation. Journal of bacteriology **188:**576-86.

3. **Feng, X., R. Oropeza, and L. J. Kenney.** 2003. Dual regulation by phospho-OmpR of ssrA/B gene expression in Salmonella pathogenicity island 2. Mol Microbiol **48:**1131-43.

4. **Fernandez-Mora, M., J. L. Puente, and E. Calva.** 2004. OmpR and LeuO positively regulate the Salmonella enterica serovar Typhi ompS2 porin gene. J Bacteriol **186:**2909-20.

5. **Gerstel, U., C. Park, and U. Romling.** 2003. Complex regulation of csgD promoter activity by global regulatory proteins. Mol Microbiol **49:**639-54.

6. **Goh, E. B., D. F. Siino, and M. M. Igo.** 2004. The Escherichia coli tppB (ydgR) gene represents a new class of OmpR-regulated genes. J Bacteriol **186:**4019-24.

7. **Guillier, M., and S. Gottesman.** 2006. Remodelling of the Escherichia coli outer membrane by two small regulatory RNAs. Molecular microbiology **59:**231-47.

8. **Higashitani, A., Y. Nishimura, H. Hara, H. Aiba, T. Mizuno, and K. Horiuchi.** 1993. Osmoregulation of the fatty acid receptor gene fadL in Escherichia coli. Mol Gen Genet **240:**339-47.

9. **Jubelin, G., C. V. Chavez, F. Taieb, M. J. Banfield, A. Samba-Louaka, R. Nobe, J. P. Nougayrede, R. Zumbihl, A. Givaudan, J. M. Escoubas, and E. Oswald.** 2009. Cycle inhibiting factors (CIFs) are a growing family of functional cyclomodulins present in invertebrate and mammal bacterial pathogens. PLoS One **4:**e4855.

10. **Maeda, S., K. Takayanagi, Y. Nishimura, T. Maruyama, K. Sato, and T. Mizuno.** 1991. Activation of the osmoregulated ompC gene by the OmpR protein in Escherichia coli: a study involving synthetic OmpR-binding sequences. J Biochem (Tokyo) **110:**324-7.

11. **Oropeza, R., C. L. Sampieri, J. L. Puente, and E. Calva.** 1999. Negative and positive regulation of the non-osmoregulated ompS1 porin gene in Salmonella typhi: a novel regulatory mechanism that involves OmpR. Mol Microbiol **32:**243-52.

12. **van Helden, J.** 2003. Regulatory sequence analysis tools. Nucleic Acids Res **31:**3593-6.

13. **Yamamoto, T., and I. Taneike.** 2000. The sequences of enterohemorrhagic Escherichia coli and Yersinia pestis that are homologous to the enteroaggregative E. coli heat-stable enterotoxin gene: cross-species transfer in evolution. FEBS Lett **472:**22-6.

14. **Yoshida, T., L. Qin, L. A. Egger, and M. Inouye.** 2006. Transcription regulation of ompF and ompC by a single transcription factor, OmpR. The Journal of biological chemistry **281:**17114-23.
