# Supplementary material for: Phenotypic and transcriptional analysis of the osmotic regulator OmpR in Yersinia pestis
Source: BMC Microbiol. 2011 Feb 23;11:39. doi: 10.1186/1471-2180-11-39 (PMC3050692; doi:10.1186/1471-2180-11-39)
Supplement: Additional file 4 — The 16 verified OmpR-dependent genes. [file 1471-2180-11-39-S4.DOC]

**Verified OmpR-dependent genes under osmotic stress**

| **Gene ID** | **Gene** | **Product** | **Regulation** | **Microarray** | **RT-PCR** | **Computational marching of the OmpR consensus** | | |
| --- | --- | --- | --- | --- | --- | --- | --- | --- |
|  |  |  |  |  |  | **Position§** | **Sequence** | **Score** |
| **The 14 genes disclosed by microarray** | | | | | | | | |
| YPO0509 |  | hypothetical protein | + | -7.56 | -6.88 | D-28...-9 | TTAATATCTGGAAACATTTT | 8.42 |
| YPO0608 |  | membrane protein | - | 53.28 | 9.85 | R-145...-126 | AAAACATTTTATTACATTTA | 9.08 |
| YPO0609 |  | transporter ATP-binding protein | - | 33.01 | 12.13 | D-197...-178 | AAAACATTTTATTACATTTA | 9.08 |
| YPO1222 | *ompC* | outer membrane porin protein C | + | -9.68 | -10.96 | D-185...-166 | ATAAATACTTGTTGCAATTT | 7.06 |
| YPO1411 | *ompF* | outer membrane porin protein F | + | -24.28 | -18.38 | D-194...-175 | TTTACATTTTGTAACACATA | 11.57 |
| YPO1634 | *phoP* | response regulator protein | + | -3.03 | -3.12 | R-177...-158 | TTTAAATGTTGTAACAATTT | 8.54 |
| YPO1683 |  | N-acetylmuramoyl-L-alanine amidase | + | -2.42 | -2.97 | D-38...-19 | TTAACACTTAAATTCATTTT | 7.87 |
| YPO2155 |  | exported protein | + | -2.21 | -4.09 | R-26...-7 | AATTCCCTTAGTAACAAAAT | 7.68 |
| YPO3034 | *maeB* | NADP-dependent malic enzyme | + | -3.25 | -2.69 | R-124...-105 | CATACTCTGCGTAACATTTT | 7.98 |
| YPO3512 | *rplU* | 50S ribosomal protein L21 |  | 2.61 | 1.49 | R-151...-132 | AGCACAGTTTATTACATCTC | 8.07 |
| YPO3707 |  | conserved hypothetical protein | + | -3.20 | -2.57 | R-287...-268 | GATCAGTTTTGTAACATTTG | 7.28 |
| YPO3708 |  | conserved hypothetical protein | + | -4.10 | -3.74 | D-256...-237 | GATCAGTTTTGTAACATTTG | 7.28 |
| YPO4018 | *cysM* | pyridoxal-phosphate dependent protein | - | 39.04 | 3.31 | R-239...-220 | TTTACCCATGATTTCATCTT | 8.02 |
| YPO4020 |  | membrane protein | - | 84.93 | 3.34 | R-288...-269 | TGTACTCAACGTATCATCTA | 7.33 |
| **Additional two genes disclosed by further biochemical assays** | | | | | | | | |
| YPO0136 | *ompR* | transcriptional regulator OmpR | + | ND | ND | D-113...-94 | AATAAGCTTTGTAACAATTT | 10.34 |
| YPO2506 | *ompX* | outer membrane protein X | - | 2.50 | -2.97 | R-125...-106 | GAAATTCTTTGTTACATGAA | 6.03 |

§, The numbers indicated the nucleotide position upstream the start code

+ and – indicated positive and negative regulation by OmpR, respectively.
